# Nutritional status of Saudi obese patients undergoing laparoscopic sleeve gastrectomy, one-year follow-up study

**DOI:** 10.1017/S0007114524002460

**Published:** 2024-12-14

**Authors:** Seham J. Alqahtani, Hanan A. Alfawaz, Fuad A. Awwad, Ahmad T. Almnaizel, Anwar Alotaibi, Adnan S. Bajaber, Afaf El-Ansary

**Affiliations:** 1 Department of Food Science & Nutrition, College of Food & Agriculture Sciences, King Saud University, Riyadh, Saudi Arabia; 2 Quantitative Analysis Department, College of Business Administration, King Saud University, Riyadh, Saudi Arabia; 3 Research Office, Johns Hopkins, Aramco Healthcare, Dhahran, Saudi Arabia; 4 Central Research Laboratory, Female Campus, King Saud University, Riyadh, Saudi Arabia

**Keywords:** Bariatric surgery, Vitamin deficiencies, Sleeve gastrectomy, Nutritional status

## Abstract

Bariatric surgery has significantly increased globally as an effective treatment for severe obesity. Nutritional deficits are common among candidates for bariatric surgery, and follow-up of nutritional status is critically needed for post-surgery healthcare management. This observational prospective study was conducted at King Khalid University Hospital in Riyadh. Samples were collected pre- and post-laparoscopic sleeve gastrectomy (LSG), with the visit intervals divided into four visits: pre-surgery (0M), 3 months (3M), 6 months (6M) and 12 months (12M). Food intake and eating patterns significantly changed during the first year (*P* < 0·001). The mean energy intake at 3M post-surgery was 738·3 kcal, significantly lower than the pre-surgery energy intake of 2059 kcal. Then, it increased gradually at 6M and 12M to reach 1069 kcal (*P* < 0·00). The intake of Fe, vitamin B_12_ and vitamin D was below the dietary reference intake recommendations, as indicated by the 24-hour dietary recall. The prevalence of 25 (OH) vitamin D deficiency improved significantly from pre- to post-surgery (*P* < 0·001). Vitamin B_12_ deficiency was less reported pre-LSG and improved steadily towards a sufficient post-surgery status. However, 35·7 % of participants were deficient in Fe status, with 28·6% being female at higher levels than males. While protein supplementation decreased significantly over the 12M follow-up, the use of vitamin supplements dramatically increased at 3 and 6M before declining at 12M. Fe and vitamin B_12_ were the most popular supplements after vitamin D. This study confirms the necessity for individualised dietary plans and close monitoring of candidates’ nutritional status before and after bariatric surgery.

Obesity, defined as abnormal or excessive fat accumulation posing health risks, is globally prevalent and is on the rise, particularly in economically affluent regions such as Western Europe, the USA and the Gulf Cooperation Council^(1,2)^ Based on the BMI, the classification considers individuals with a BMI of at least 30 kg/m^2^ as obese. According to the WHO and the Global Obesity Observatory, Gulf Cooperation Council countries have obesity rates that vary considerably. These rates range from 27·8 % to 43·7 %^(3,4)^. In Saudi Arabia, the World Health Survey reported a 20·2 % obesity prevalence in 2019, increasing to approximately 25 % by 2020^(5,6)^. Obesity management primarily focuses on lifestyle improvements and medical interventions^(7)^. Early treatment for overweight individuals highlights practices including controlling and maintaining a balanced diet by reducing high-energy and overeating habits while increasing organic and high-fibre food intake and engaging in consistent physical activities to align dietary intake with energetic expenditure^(8)^. However, for patients with morbid obesity (BMI ≥ 40·09 kg/m^2^), lifestyle improvements and coaching may be insufficient, necessitating medical interventions^(7,8)^. Treatments for morbid obesity include pharmacological approaches to support weight loss maintenance and bariatric surgery, which aim to reduce stomach size^(7,9)^. Consequently, the effective management of morbid obesity often involves a combination of lifestyle adjustments and medical interventions targeting the size and composition of the digestive system^(7)^.

Bariatric surgery is recommended for individuals with resistant obesity, specifically those with a BMI ≥35 kg/m^2^, regardless of presence, absence or severity of co-morbidities. Bariatric surgery should be considered for individuals with a BMI of 30–34·9 kg/m^2^ with severe comorbidities, such as hypertension, hyperlipidaemia and type II diabetes^(10)^. Laparoscopic sleeve gastrectomy (LSG) is the preferred global procedure, followed by Roux-en-Y gastric bypass (RYGB)^(11)^. LSG involves the removal of the fundus and greater curvature of the stomach, resulting in an 80 % reduction in stomach capacity^(12)^. While Bariatric surgery effectively stimulates weight loss, it leads to the risk of nutritional deficiencies, including Fe, fat-soluble vitamins (mainly A and D) and some water-soluble vitamins, as well as nutritional status, worsening if there are any deficiencies before surgery^(13)^. Although nutrient deficiencies are commonly found in RYGB patients, comparable rates of long-term nutritional deficiencies have been determined in LSG patients^(13,14)^. Several factors influence the long-term health of bariatric surgery patients, including their individual response to the surgery and their ability to adopt healthy lifestyle habits^(15)^. According to research, patients demonstrate the most significant commitment to food and lifestyle recommendations pre- and directly post-surgery; nevertheless, as the month progresses, the rate of adherence declines^(15)^.

Medical records indicate that LSG is the most commonly performed bariatric procedure in Saudi Arabia, followed by RYGB and gastric banding^(16)^. LSG is favoured for its perceived simplicity and potential safety advantages over RYGB, contributing to its higher regional prevalence^(17)^. Despite a growing body of research on the post-surgery effects of bariatric procedures, there is a need to delve into the preoperative nutritional status of patients, particularly in Gulf Cooperation Council‘^(14)^. Given the expanding use of bariatric surgery in Saudi Arabia, examining nutritional status pre- and post-bariatric surgery is critical to offer insight into the present nutritional state. To our knowledge, prospective cohort studies on the nutritional status of candidates for bariatric surgery are scarce in Saudi Arabia. This study aimed to determine the amount of pre- and post-surgery nutrient deficiencies in bariatric candidates who underwent LSG and to evaluate the evolution of nutritional status during the first postoperative year. A comprehensive understanding of a patient’s health allows healthcare teams to tailor pre- and post-bariatric surgery care, ultimately improving patient outcomes.

## Materials and methods

### Study participants

Obese participants aged 19–60 years were recruited according to the international criteria of bariatric surgery: BMI > 40 kg/m^2^ or > 35 kg/m^2^ with some comorbidities according to the Saudi Arabian Society of Metabolic and Bariatric Surgery guidelines for the prevention and management of obesity in SA^(18)^. Recruitment started from January 2021 to July 2021 at the King Khalid University Hospital, Riyadh, SA. All patients who visited the bariatric clinic at that time were screened for study eligibility and who matched the inclusion criteria and were accepted to participate were included in this study.

### Inclusion criteria

Participants were ≥ 18 years old and were not pregnant or breast-feeding. Participants had no history of major gastrointestinal surgery and were free of food allergies, colon cancer, inflammatory bowel disease or chronic diarrhoea within the past 2 months. Also, participants were not receiving any of the following: antibiotics, pre- or probiotic agents within 3 months before fecal sampling.

Thirty-four participants (nineteen females and fifteen males) matched the criteria and were included in the study. However, six participants were excluded because they did not undergo surgery due to hyperglycaemia (three participants), pregnancy (one participant) or contracting COVID-19 (one participant). In addition, (1 participant) was lost to follow-up. Detailed participant information can be found in our recently published study^(19)^.

### Study design and data collection

An observational prospective study was conducted as part of a bariatric surgery cohort study done for 12 months at King Khalid University Hospital in Riyadh, SA. Clinical and biological samples were obtained pre- and post-LSG, where the visit interval was divided into four visits: pre-surgery (0M), 3 months (3M), 6 months (6M) and 12 months (12M) post-surgery. All participants were interviewed face-to-face to complete a questionnaire. The questionnaire contained demographic data, including age, sex, education level, marital status and monthly income. Another section also included exercise type, time, frequency and vitamin supplementation. Anthropometric data (weight, height, waist circumference and hip circumference) were recorded during each visit. Weight was recorded to the nearest 0·1 kg with light clothing and without shoes using an international standard scale (Digital Pearson Scale; ADAM Equipment Inc.). Height was recorded using the same scale to the nearest 0·5 cm while standing without shoes and facing the scale. Waist circumference was measured at the narrowest level between the lowest rib and umbilicus, where the normal ranges were < 94 cm for male and < 80 cm for female, and hip circumference (HC) was measured at the level of the great trochanter, with the legs close together using non-stretchable tape^(20)^. Both the measurements were recorded to the nearest 0·5 cm. The waist:hip ratio was calculated by dividing the waist circumference by the HC, where the cut-off < 0·90 and < 85 are considered ‘normal’ waist:hip ratio for males and females, respectively^(20)^.

### Dietary data

Dietary intake data were measured by a 24-hour recall three times at every visit (two consecutive days on the weekdays and one day on the weekend) to obtain detailed information about energy and nutrients. In addition, the FFQ included questions about fruits, vegetables, dairy, meat, sweet food, sweetened drinks, the number of main and snack meals, fast food and savory food. The answers were always, often, sometimes, rarely and never. The FFQ was validated by piloting it to 100 bariatric patients. Then, an internal consistency test by Pearson correlation and a reliability test using Cronbach’s alpha were performed, and both were statistically significant.

These data were measured at each visit over 12M follow-up. Instructions were given to participants on how to recall all food intake, and they were then asked to recall the quantity of food consumed. Food models (Fort Atkinson), household units (e.g., bowls, spoons and cups) and photographic pictures (Nelson, MAFF) were used to estimate food portion size. Study participant entries were reviewed for accuracy and entered into a nutrition analysis software program (Food Processor 10·15; Esha Research). The participants who underwent LSG did not receive any pre-surgery diet. However, a nutrition orientation session was only held to provide advice and recommendations regarding food intake, supplementation, diet stages and adherence to dietary and lifestyle recommendations post-surgery. Post-surgery diet progression followed a gradual return to solid foods (liquids, pureed, soft solids and regular foods) within a maximum of 8 weeks, as directed by their doctors. After 3 months, they scheduled a dietitian clinic for nutritional counseling to follow-up with any patient inquiries and to provide more supplementation if needed.

### Clinical chemistry data

Blood samples were obtained and analysed according to the standard laboratory protocols employed by the hospital for routine analysis. The reference ranges adopted for this study were as follows: total protein: 60–80, vitamin D, 50–175 nmol/l; vitamin B_12_, 156–677 pmol/l, Fe: 11–31·3 umol/l. Nutritional deficiency or excess levels were defined as concentrations below or above the reference range of the hospital laboratory.

### Statistical analysis

All statistical analyses were performed using the statistical software R, version 4·2·1. Statistical significance was set at *P* < 0·05. Continuous variables are summarised using means and standard deviations or medians and interquartile ranges based on normality. Categorical variables are summarised as frequencies and percentages. Repeated measures analysis of variance ANOVA with pairwise comparisons and corrections for sphericity violations was used to assess differences in continuous outcomes over time. Generalised estimating equation models with binomial distribution and logit link function were used to analyse the predictors of supplement use and exercise over time, while accounting for within-subject correlations. Spearman’s rank correlation analysis was used to evaluate the association between supplement use and nutrient levels at different time points. Heat maps visually depict the correlation strengths.

## Results

### Demographic characteristics

Among the twenty-eight participants, 53·57 % were female and 46·43 % were male. Most participants fell into the age range of 19–30 years (53·57 %). Most participants (71·43 %) were in a healthy status without any comorbidities. The additional details are provided in Table 1.

**Table 1.** The characteristics of participants

Participants (no.)	28	*n*	%
Gender	Male	13	46·4
	Female	15	53·6
Age group: (year)	19–30	15	53·6
	31–40	5	17·9
	41–50	5	17·9
	> 50	3	10·7
Marital status	Single	12	42·9
	Married	15	53·6
	Divorced	1	3·6
Education level	Primary or less	2	7·1
	Secondary	9	32·1
	Graduate	15	53·6
	Postgraduate	2	7·1
Income level: (Saudi Riyal)	< 5000	9	32·1
	5000–10 000	9	32·1
	>10 000–15 000	5	17·9
	>15 000–20 000	2	7·1
	>20 000	3	10·7
Medical history	Healthy	20	71·4
	Sleep apnea	3	10·7
	Hypertension	2	7·1
	CVD	1	3·6
	DM, HTN.	1	3·6
	DM, HTN, high cholesterol	1	3·6

HTN, hypertension; DM, diabetes mellitus.

### Anthropometric and lifestyle characteristics

Anthropometric measurements changed significantly during the 12M following the surgery (*P* < 0·05). The changes in participants’ analyses were significant *P* < 0·05 in weight, BMI, Hip, and WC, except for waist:hip ratio (Table 2). The LSG resulted in a sharp and significant body weight loss from M0 121·31 kg ± 16·35 to M3 weight 98·70 kg ± 13·52 as they lost 23 kg in the first three months post-surgery. Participants continued to lose weight significantly at M6 (87·23 kg ± 12·73) by losing an extra 11·47 kg from 3 to 6 months after surgery. After that, the body weight decreased slowly during the next 6 months until they reached 80·70 kg ± 11·65 with only 6·53 kg weight loss from M6 to M12 (Table 2). For most variables, improvement occurred rapidly and significantly in the first 3–6 months post-surgery. At 12 M, the participants had lost (33 %) of their initial weight.

**Table 2.** Changes of anthropometrics among the participants over 12 months pre- and post-surgery

Characteristics	0M		3M		6M		12M		*P* value
	Mean	sd	Mean	sd	Mean	sd	Mean	sd	
Weight (kg)	121·31	16·35	98·70	13·52	87·23	12·73	80·70	11·65	<0·001^**^
BMI (kg/m^2^)	44·07	5·25	36·01	5·47	31·79	5·96	29·40	5·87	<0·001^**^
Waist circumference (WC) (cm)	125·82	12·56	110·02	11·25	101·25	8·53	97·13	8·51	<0·001^**^
Hip circumference (HC) (cm)	141·50	19·20	123·14	10·20	115·07	10·50	110·25	10·80	<0·001^**^
Waist:hip ratio (WHR)	0·89	0·10	0·89	0·10	0·88	0·08	0·89	0·09	<0·631
Percentage of excess weight loss (%EWL)	–		46·01	18·20	70·55	31·02	82·69	30·60	<0·001^**^
Percentage of total weight loss (%TWL)	–		18·53	4·72	27·81	7·82	33·00	9·11	<0·001^**^

** Variables considered significant if *P* value <0·001.

Considering participants’ vitamin supplementation use, the majority of the participants did not use multivitamins (89·3 %), vitamin D (75 %) or vitamin B_12_ (78·6 %) pre-operatively. However, the use of vitamins post-surgery increased significantly at 3M and 6M, then decreased at 12M *P* < 0·001) (S. Table A1). No significant differences were found between males and females in supplements used pre- or post-operatively.

Considering the participants’ exercise, only 35·7 % of participants reported engaging in exercise pre-surgery. This number significantly increased to 89·3 % by 12M post-surgery (*P* < 0·001). Walking was the most activity performed at all times, with time increased post-surgery compared with pre-surgery (*P* < 0·001) (S. Table A2).

The results of the generalised estimating equation are shown in online Supplementary Table 3. These models predicted the likelihood of supplement use and exercise over time. The analysis revealed that, for variables such as multivitamins, vitamin D, vitamin B_12_, Ca and Fe, time significantly affected the likelihood of supplement use. Specifically, for multivitamins, the OR was 2·46 (95 % CI: 1·53, 3·97, *P* < 0·001), indicating a significant increase in the likelihood of use with each unit increase in time. Similarly, for vitamin D, the OR was 1·92 (95 % CI: 1·24, 2·97, *P* < 0·004), for vitamin B_12_, the OR was 1·79 (95 % CI: 1·17, 2·74, *P* < 0·007), for Ca, the OR was 1·61 (95 % CI: 1·15, 2·24, *P* < 0·005) and for Fe, the OR was 1·97 (95 % CI: 1·36, 2·85, *P* < 0·001). However, the protein supplementation time did not significantly affect this likelihood.

Exercise also showed a significant positive association with time, with an OR of 2·68 (95 % CI: 1·59, 4·53, P < 0·001). This indicates the likelihood of engaging in exercise increased with each time point.

### Clinical data

**Fig. 1.**
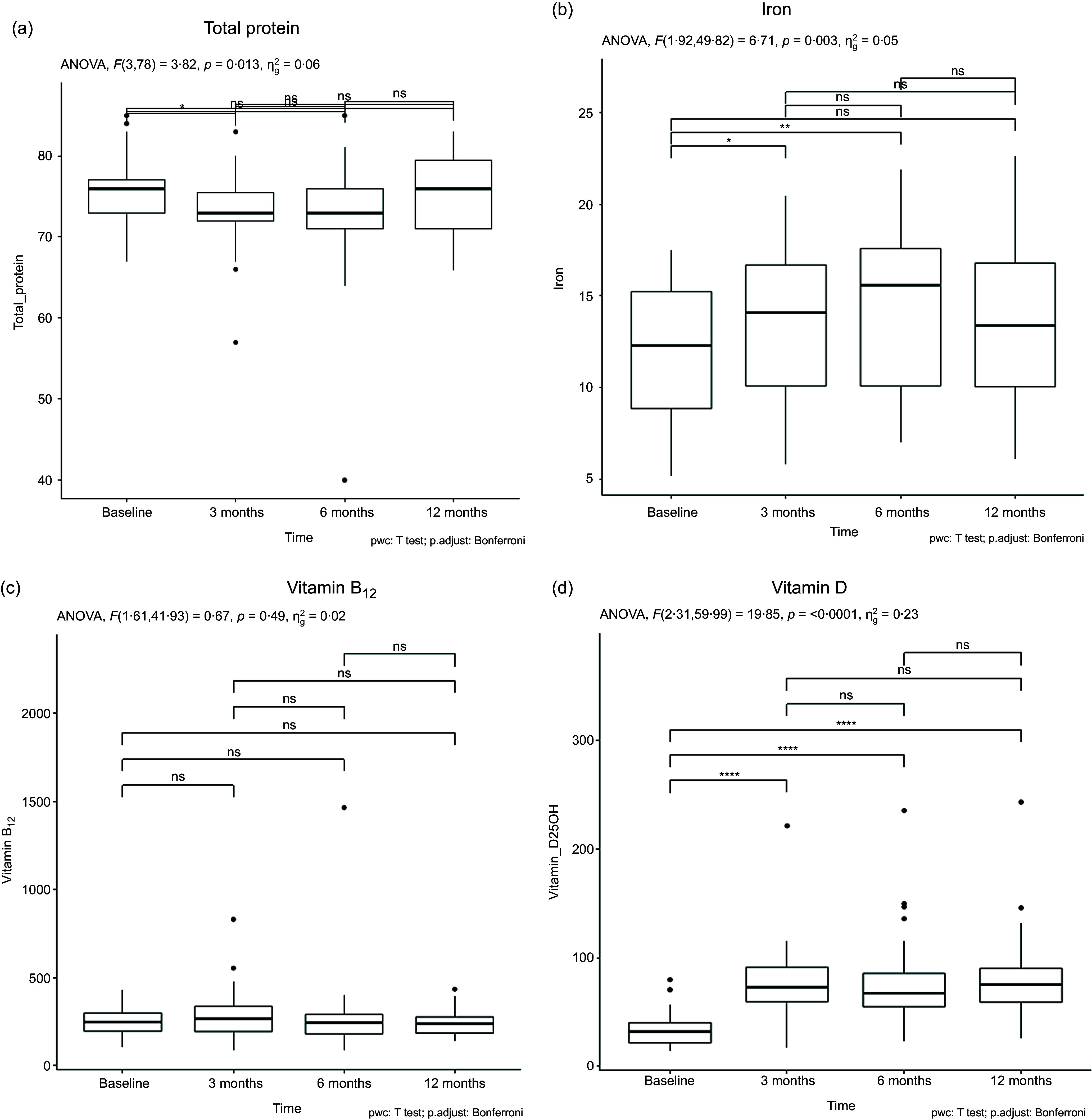
Changes of nutrient level in blood among the participants over 12 months pre- and post-surgery, (a) total protein, (b) iron, (c) vitamin B_12_ and (d) vitamin D.

Repeated-measures ANOVA and *post hoc* findings for biochemical markers are outlined in Fig. 1. The total protein and Fe levels showed significant changes over time (*P* < 0·013 and *P* < 0·003, respectively). *Post hoc* tests revealed significant differences between specific time points for both the variables. No significant changes were observed in vitamin B_12_ levels (*P* = 0·488). Vitamin D25OH levels exhibited significant variation over time (*P* < 0·001), except for a specific pairwise group comparison.

**Fig. 2.**
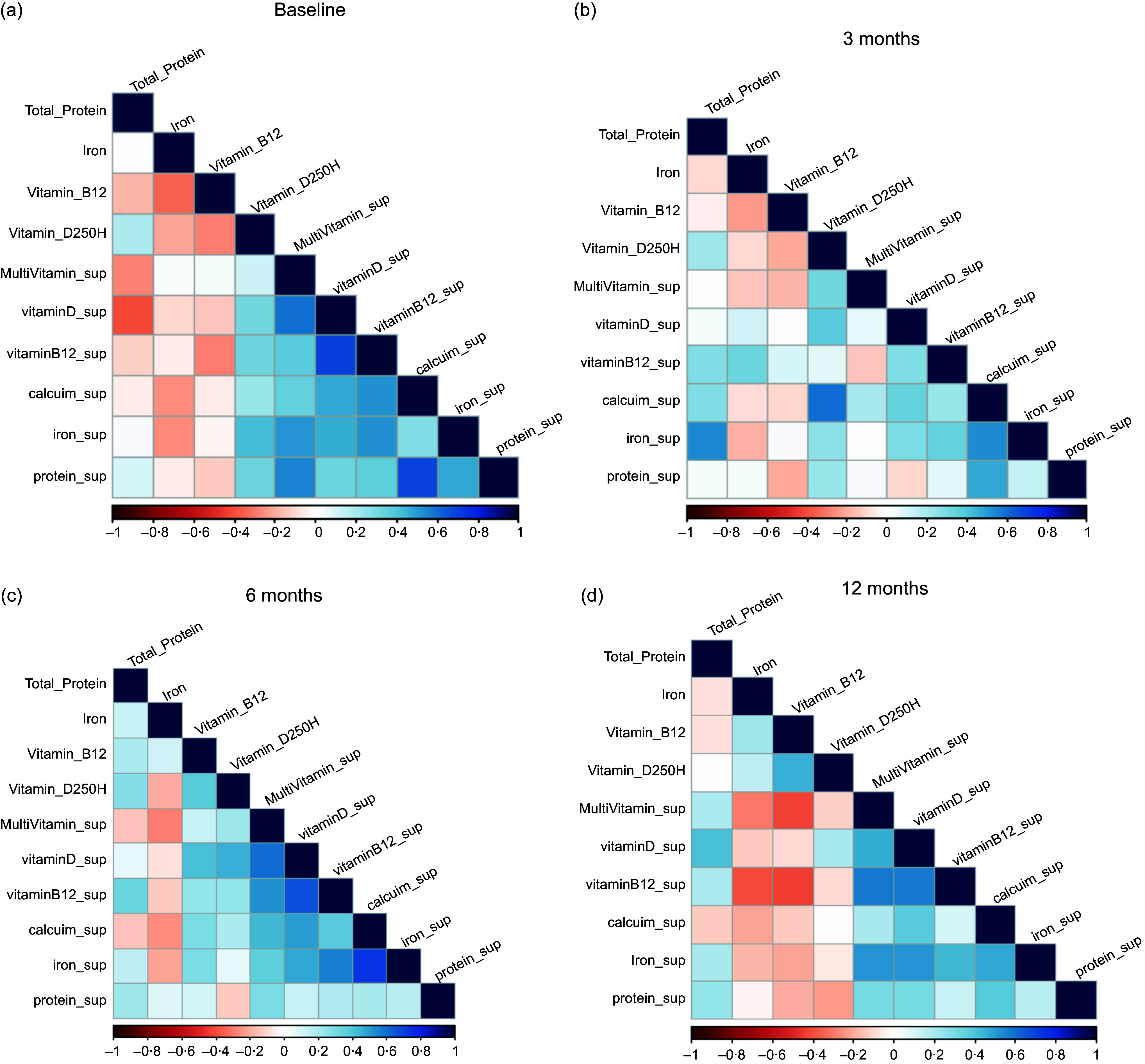
The heat maps show how supplemental nutrient levels correlated with measured blood parameters over time (a) baseline, (b) 3 months, (c) 6 months and (d) 12 months.

The heat maps provide insight into how supplemental nutrient levels correlate with measured blood parameters over time (Fig. 2). At baseline, higher vitamin D supplement use was correlated with higher vitamin D levels in the blood (*r* = 0·31). Ca supplement intake also positively correlated with vitamin D levels (*r* = 0·36). By 3 months, Fe levels had increased slightly as vitamin D supplement use increased (*r* = 0·11). The total protein levels also increased with increased Fe supplement intake (*r* = 0·54). At 6 months, strong positive correlations emerged between vitamin B_12_ supplement use and actual vitamin B_12_ levels (*r* = 0·42). Higher multivitamin intake was correlated with increased vitamin D levels (*r* = 0·61). Notably, several supplement-lab correlations were strengthened at 12 months. The vitamin D supplement dosage correlated strongly with vitamin D levels (*r* = 0·57). A similar correlation was observed between B_12_ supplementation and B_12_ levels (*r* = 0·57). Higher multivitamin intake correlated with higher levels of B_12_, D and Fe. Total protein was also positively correlated with multivitamins (*r* = 0·20) and vitamin D supplement amounts (*r* = 0·42). These dynamic relationships suggest that supplemental regimens increasingly impacted laboratory results over time. The positive correlations indicated that supplements generally raised the associated nutrient blood levels, as intended.

### Nutritional deficiency

**Fig. 3.**
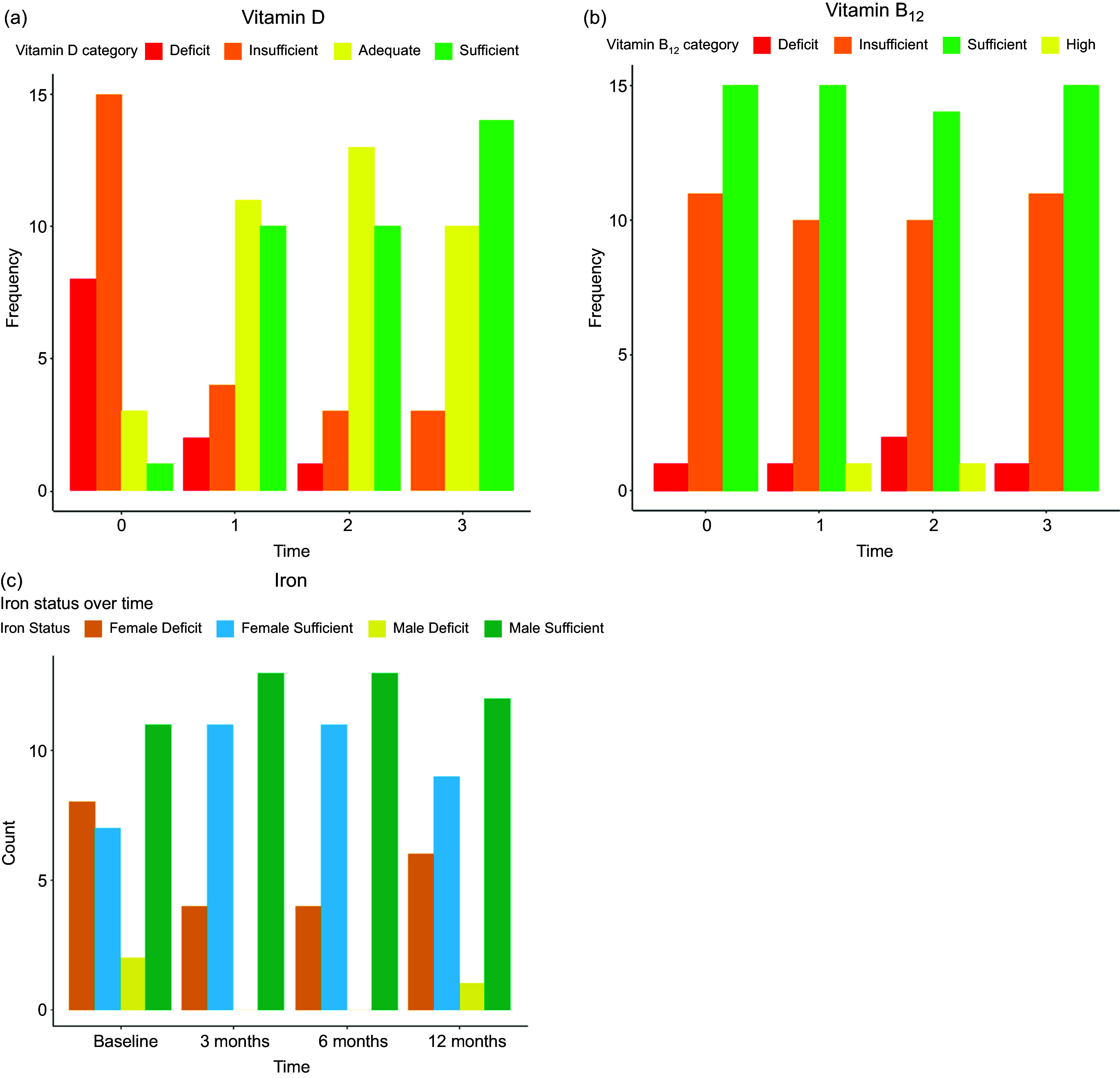
Prevalence of nutrient deficiencies among the participants over one-year follow-up (a) vitamin D, (b) vitamin B_12_ and (c) iron.

The distributions of vitamin D, B_12_ and Fe status at different time points (baseline, 3 months, 6 months and 12 months) are illustrated in Fig. 3 and Tables 3–5. For vitamin D, at the baseline, 32·1 % had a vitamin D deficit, 53·6 % had insufficient levels, 10·7 % had adequate levels and 3·6 % had sufficient levels. Over time, there was a decrease in the percentage of participants with deficit and insufficient levels and an increase in those with adequate levels. By 12 months, no participants had a deficit, with 10·7 % having insufficient levels, 35·7 % having adequate levels and 53·6 % having sufficient levels. For vitamin B_12_, at baseline, 3·6 % had a vitamin B_12_ deficit, 42·8 % had insufficient levels and 53·6 % had sufficient levels. None of the participants had a high score. Over time, there was a decrease in the percentage of participants with deficits and an increase in those with sufficient levels of deficits. By 12 months, the percentage of participants with deficits decreased to 3·6 %, with 39·3 % having insufficient levels and 57·1 % having sufficient levels. None of the participants had a high score.

**Table 3.** Prevalence of vitamin D deficiency over one-year follow-up

Vitamin D status	Reference range (nmol/l)	0M		3M		6M		12M	
		*n*	%	*n*	%	*n*	%	*n*	%
Deficit	< 25	9	32·1	2	7·1	1	3·6	0	
Insufficient	25–50	15	53·6	4	14·3	3	10·7	3	10·7
Adequate	50–75	3	10·7	11	39·3	13	46·4	10	35·7
Sufficient	75–250	1	3·6	11	39·3	11	39·3	15	53·6
	Total	28	100·0	28	100·0	28	100·0	28	100·0

**Table 4.** Prevalence of vitamin B_12_ deficiency over one-year follow-up

Vitamin B_12_ status	Reference range (pmol/l)	0M		3M		6M		12M	
		*n*	%	*n*	%	*n*	%	*n*	%
Deficit	<148	1	3·6	1	3·6	3	10·7	1	3·6
Insufficient	148–221	12	42·9	11	39·3	10	35·7	11	39·3
Sufficient	221–570	15	53·6	15	53·6	14	50·0	16	57·1
High	>570	0		1	3·6	1	3·6	0	
	Total	28	100·0	28	100·0	28	100·0	28	100·0

**Table 5.** Prevalence of iron deficiency over one-year follow-up

			0M		3M		6M		12M	
Fe status		References range (nmol/l)	*n*	%	*n*	%	*n*	%	*n*	%
Deficit	Male	<11·5	2	7·1	0		0		1	3·6
	Female	<9	8	28·6	4	14·3	4	14·3	6	21·4
Sufficient	Male	11·6–31·3	11	39·3	13	46·4	13	46·4	12	42·9
	Female	9–30·4	7	25	11	39·3	11	39·3	9	32·1
		Total	28	100·0	28	100·0	28	100·0	28	100·0

### Dietary intake from 24 h food recall and FFQ

**Fig. 4.**
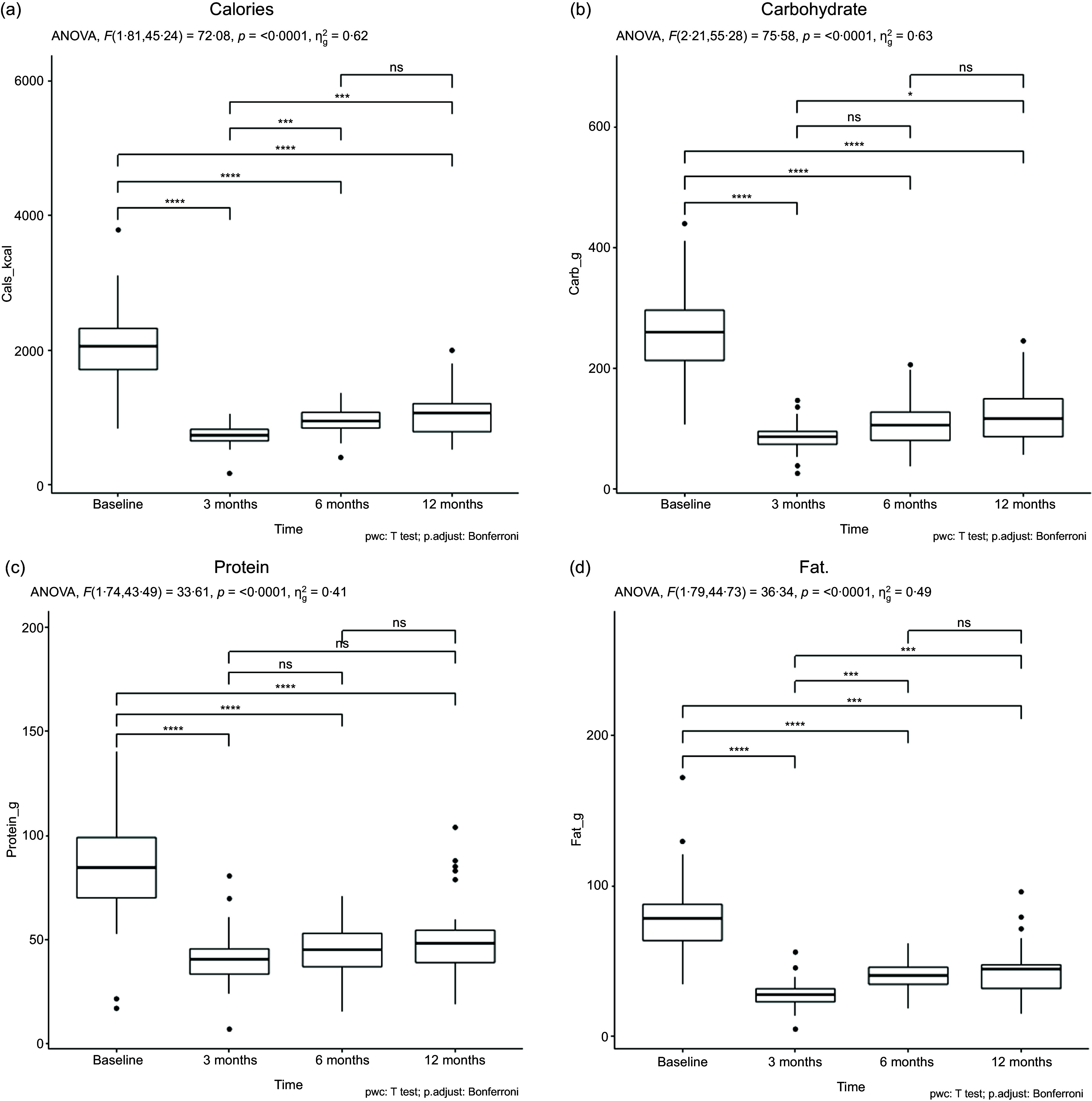
Calories and macronutrients values in participants over 12 M follow-up from 24-hour dietary recall. (a) Calories, (b) carbohydrate, (c) protein and (d) fat.

Figure 4 and online Supplementary Fig. 1 show the ANOVA results for the dietary intake variables. The analysis revealed significant effects of time on 24-hour food recall. For energy intake, carbohydrate intake, protein intake, fat intake (Fig. 4(a)–(d)), saturated fat intake, cholesterol intake, total fibre intake, sugar intake and vitamin D intake (S. Figure A1), there were significant differences between most time points, except for the comparisons between 6 and 12 months, which were not statistically significant. Water intake and vitamin B_12_ intake did not show significant differences between specific time points. Ca intake showed a significant effect of time, with significant differences observed between baseline and 6 months and between baseline and 12 months, but not between the other time point comparisons (details shown in S. Figures A1). The mean energy intake at 3M post-surgery was 738·3 kcal, which was significantly lower than the pre-surgery energy intake of 2059 kcal. Then, it increased gradually at 6M and 12 M to reach 1069 kcal, which was statistically significant compared with the pre-surgery value (*P* < 0·00). Pre-surgery, mean fat, carbohydrate and protein intake were 78·36 ± 31·41 g/d, 259·98 ± 83·81 g/d and 84·69 ± 31·814 g/d, respectively, which were above the dietary reference intake recommendations^(21)^. The intake of Fe, vitamin B_12_ and vitamin D was found to be under the dietary reference intake recommendations (S. Figures A1).

The results from the FFQ showed that 17·9% of participants always consumed sweetened drinks, 28·6% consumed them 3–4 times a week during the last month pre-surgery and 28·6% reported consuming them rarely. In addition, 14·3%, 35·7% and 17·9% of participants reported eating sweet food always, sometimes and rarely, respectively. 96·7 % and 85·4 % of participants rarely or never consumed sweetened drinks or sweet food at 3M post-surgery and continued with a comparable pattern over 12M (S. Table A5). Participants’ eating habits went towards healthy choices as their habits of eating fast food or in restaurants dropped significantly over 1 year after surgery (*P* < 0·001) (S. Table A5). Furthermore, in the present study, the number of meals and snacks during the day increased significantly (*P* < 0·013 and 0·024, respectively) to reach six meals daily, three snacks and three main meals.

## Discussion

### Nutritional deficiency

Pre- and post-nutritional evaluation and monitoring are crucial for identifying potential deficiencies and making necessary corrections^(22,23)^. This study highlighted the importance of nutritional management pre and post-bariatric surgery. Several studies from different countries have defined nutritional status pre- and post-LSG over one year^(24–26)^. Unlike most available studies, this was a prospective study following the participant for one year post-surgery, which provided a clear perspective on nutritional status among surgical candidates.

Participants often suffer from deficiencies in pre-surgery due to unhealthy diets and unbalanced nutritional compositions^(26–28)^. The present study found that people with obesity often have 25(OH) vitamin D deficiency. This could result from insufficient consumption, an indoor sedentary lifestyle without sun exposure and vitamin D sequestration in adipose tissue^(29,30)^. This deficiency is influenced by genetic variation and can lead to impaired absorption of vitamin D3^(28)^. In the present study, the prevalence of 25(OH) vitamin D deficiency continued pre-and post-surgery, with a significant increase in mean serum 25(OH) vitamin D levels over a one-year follow-up *P* < 0·001). This is consistent with previous studies showing that vitamin D deficiency is a prevalent issue pre- and post-LSG, with deficiency improving from 46 to 14 %^(31)^ and from 84·62 to 48 %^(32)^ over a one-year follow-up. While there is no agreement on ideal vitamin D concentrations, emerging research shows that levels higher than 75 nmol/ml could be sufficient to preserve health^(33)^. The present study also found that prescribing 50 000 IU of vitamin D (2 times/month) and high adherence levels may have contributed to the lower post-surgery deficiency prevalence. In addition, it could be based on the correlation between systemic vitamin D concentrations and adipose tissue mass, which supports storage in fatty tissues and release after weight reduction^(34)^.

Metabolic surgeries such as LSG are less likely to cause vitamin B_12_ deficiency compared with RYGB surgery. The body stores vitamin B_12_, providing a reserve. While adequate pre-surgical B_12_ levels are beneficial, the LSG can lead to malabsorption, potentially causing a B_12_ deficiency over time^(26)^. The deficiency is anticipated due to the removal of the fundus, loss of intrinsic factors by parietal cells and reduction of gastric acid post-LSG^(35)^. In the present study, vitamin B_12_ deficiency was less reported pre-surgery and improved steadily towards a sufficient post-surgery status, which aligns with findings from previous studies^(22,28,36–38)^. This may be due to good adherence with supplementation, as more than 67 % of our participants were taken B_12_ supplementation over 12 months post-surgery. In addition, Zarshenas *et al.* (2016) found that adherence with prescribed supplementation prevented their participants from developing B_12_ deficiency after 12 months post-surgery^(31)^. However, Gillon *et al.* found an increasing prevalence of B_12_ deficiency in 19 % of the subjects who were deficient compared with 6·4 % preoperatively because of no adherences over 12 months post-operatively^(38)^. Thus, adherence to prescribed supplements decreases the risk and incidence of vitamin B_12_ deficiency in participants post-surgery^(39)^.

Fe deficiency is a prevalent issue in individuals with obesity before surgery, affecting 20–47 % of the population^(33,40)^. This condition is more common in younger participants and women and can lead to chronic diseases, such as anaemia and disturbance of Fe absorbance at the enterocyte level^(30)^. Persistent inflammation disrupts Fe homeostasis, possibly because of increased systemic Fe expression^(40)^. This study found that 35·7 % of participants were deficient, with 28·6 % of females having higher levels than males. The American Society of Hematology reported that 33–49 % of patients who underwent LSG were more likely to develop anaemia within 2 years^(41)^. Factors such as anatomic changes, decreased stomach acidity and long-term use of medicines that restrict gastric acid secretion can aggravate Fe deficit^(30,31,42)^. Other factors, such as decreased consumption of Fe-rich foods, dietary changes and lack of compliance with oral Fe supplementation, can also aggravate Fe deficits^(43,44)^. The study’s findings align with those of previous research, showing that Fe deficiency is more common in females than males post-surgery. A recent study in China reported that females were more likely to suffer from Fe deficiency post-surgery than men, with 43·8 *v*. 0 %, *P* < 0·001, respectively. This was probably a consequence of blood loss during menstruation^(45)^. On the other hand, Hakeam *et al.* showed that only 1·6 % developed Fe deficiency anaemia 12 months post-LSG^(46)^. This may result from excluding participants with Fe deficiency anaemia pre-surgery or providing Fe supplementation with multivitamins post-surgery^(13)^. The latest recommendation is oral Fe supplementation for all participants post-surgery, but intravenous Fe therapy is essential in difficult situations^(47)^. Adherence to supplementation pre and post-surgery may decrease the risk and incidence of Fe deficiency in surgical candidates. Fe, vitamin B_12_ and vitamin D levels are the most frequently mentioned nutritional deficiencies post-surgery. However, some participants with post-bariatric surgery still have deficiencies in vitamins and minerals, possibly due to adherence issues or differing requirements based on food consumption and pre-surgery inadequacies^(37,48)^.

### Food intake and dietary pattern laparoscopic sleeve gastrectomy

LSG decreases total energy intake, especially in the first 6 months post-surgery, fluctuating from 700 to 900 calories daily^(32,49)^. The present study found that energy intake was significantly lower at one-year post-surgery, similar to previous studies^(49,50)^. Reduced energy intake can lead to lower consumption of all macronutrients, particularly protein, Fe, vitamin B_12_ and D^(48,51,52)^. Participants were strict about eating less food due to fear of regaining weight, which affected their nutrient adequacy. Dietary recall showed significant changes in macronutrients and micronutrients, except for vitamins B_12_ and D. For example, 68 % of the participants were considered deficient as they consumed less than 60 g of protein daily over 12 months post-surgery, which was considered less than current dietary reference intake recommendations, as indicated by the 24-hour dietary recall post-LSG. To avoid malnutrition, adequate protein consumption should be estimated based on age, sex and weight. Some studies show that protein consumption post-surgery is typically lower than recommended, often near 0·5 g/kg, in the first year^(22,53)^, although participants are encouraged to eat 1–1·5 mg/kg of ideal body weight (at least 60 g/d).

Reduction in protein intake post-surgery may be attributed to the temporary tolerance of protein-rich food, low adherence to protein supplementation, pepsinogen production, gastric acid reduction and pancreatic enzymes^(49,50)^. Consistent with the findings of the present study, a longitudinal study that lasted for 5 years found that protein intake decreased significantly 1 year post-surgery, with participants reporting a dietary protein intake of less than 60 g/d^(51)^. Other studies found similar results^(22,52,53)^. However, a study by Pellitero *et al.* found a significant increase in protein intake over 1 year, possibly due to adherence to protein supplements, diverse dietary intake or the populations studied^(43)^. Consuming adequate protein post-LSG is crucial to prevent complications and undesirable consequences, such as muscle loss, hair loss, poor wound recovery, infections and protein-energy malnutrition^(14,15)^. Protein supplementations (30–35 g/d) can assist in the first few days and positively affect weight reduction and composition^(54)^. Adherence to food and lifestyle guidelines is necessary for health and weight results over time because non-adherence increases the risk of complications post-LSG. Protein, Fe, vitamin B_12_ and vitamin D deficiencies are particularly concerning post-LSG^(53,55,56)^.

Food intake and eating patterns significantly changed over the first year post-LSG (*P* < 0·001). This is consistent with a Mediterranean population study that found significant and long-term changes in nutritional habits and eating behaviour, such as an increased preference for fruits, vegetables and low-fat savory foods, compared with pre-surgery^(57)^. Post-LSG, participants often switch from high-fat and high-sugar foods to healthier foods, especially in the early phase^(58)^. Participants in the present study avoided high-fat, sugar and energy foods, with 96·7 and 85·4 % of them rarely or never consuming sweetened drinks or sweet food. This could be because LSG surgery improves satiety and makes participants more sensitive to sweet tastes^(58)^. GLP-1, released in the small intestine when sweet substances induce taste cells sensitive to sweetness, may be responsible for the noticeable sweetness post-bariatric surgery^(59)^. The present study found that the number of daily meals and snacks increased significantly, reaching six daily, three snacks and three main meals. This increase was similar to the findings of Makaronidis *et al.* (2016), who showed that regular meal frequency increased with reduced portion size owing to appetite decline and postprandial fullness improvement post-surgery^(59)^. Pre-surgery and immediately afterwards, participants showed the most remarkable adherence to nutritional and lifestyle recommendations; however, as time passed, the adherence percentage declines^(60)^.

This study has some limitations that need to be considered when interpreting the results. First, the relatively small number of participants restricts the generalisability of the findings to a larger population. A more extensive and diverse sample size would strengthen the conclusions and enable subgroup analysis to identify potential variations. Second, the observational design of the study limits its ability to establish cause-and-effect relationships between the investigated factors. Future research employing a randomised controlled trial design could provide more definitive evidence of causal links.

### Conclusions

Commitment to nutritional guidelines is essential for achieving the most substantial results and preventing nutritional and dietary problems. Individuals with obesity typically consume an unbalanced, high-energy diet that can cause them to be deficient in vitamins, minerals and other nutrients. Post-surgery, conditions worsen and obtaining enough minerals from food alone is no longer possible. Consequently, following medical and nutritional recommendations will help decrease difficulties post-surgery and support participants in attaining successful outcomes. Given the growing practice of bariatric surgery worldwide, and in Saudi Arabia in particular, the present findings support the significance of comprehensive nutrition counseling and national clinical guidelines that assess micronutrient status preoperatively as well as permanent provision of micronutrient supplementation post-surgery, in particular Fe, vitamin B_12_, vitamin D and protein.

## Supporting information

Alqahtani et al. supplementary material Alqahtani et al. supplementary material

## Supplementary material

For supplementary material/s referred to in this article, please visit https://doi.org/10.1017/S0007114524002460
