# Supplementary material for: Nutritional status of Saudi obese patients undergoing laparoscopic sleeve gastrectomy, one-year follow-up study
Source: Br J Nutr. 2024 Nov 8;132(11):1454–65. doi: 10.1017/S0007114524002460 (PMC11660312; doi:10.1017/S0007114524002460)
Supplement: Alqahtani et al. supplementary material [file S0007114524002460sup001.docx]

**Appendix**

**Table A1.** Supplementation use among participants over 12 months post-surgery.

| Supplement | **Baseline** | | **3 months** | | **6 months** | | **12 months** | |
| --- | --- | --- | --- | --- | --- | --- | --- | --- |
|  | n | % | n | % | n | % | n | % |
| Multivitamin | 3 | 10.70 % | 23 | 82.10% | 24 | 85.70% | 21 | 75.00% |
| Vitamin D | 7 | 25 % | 24 | 85.70% | 23 | 82.10% | 20 | 71.40% |
| VitB12 | 6 | 21.40 % | 26 | 92.80% | 22 | 78.60% | 19 | 67.90% |
| Calcium | 2 | 7.20 % | 12 | 42.90% | 14 | 50.00% | 11 | 39.30% |
| Iron | 4 | 14.30 % | 17 | 60.70% | 19 | 67.90% | 18 | 64.30% |
| Protein | 1 | 3.60 % | 17 | 60.70% | 9 | 32.10% | 5 | 17.90% |
| Omega3,6 | 2 | 7.10 % | 1 | 3.60% | 1 | 3.60% | 3 | 10.70% |

**Table A2**: Exercise frequency, type, and duration among participants over 12 months post-surgery.

| **Exercise** | **Baseline** | | **3 months** | | **6 months** | | **12 months** | |
| --- | --- | --- | --- | --- | --- | --- | --- | --- |
|  | n | % | n | % | n | % | n | % |
| Exercise Frequency | 10 | 35.7 % | 22 | 78.7 % | 26 | 85.70% | 25 | 89.3 % |
| No Exercise | 18 | 64.3 % | 6 | 21.4 % | 2 | 7.1 % | 3 | 10.7 % |
| Walking | 8 | 28.6 % | 20 | 71.4 % | 17 | 60.7 % | 19 | 67.90% |
| Cardio & resistance | 2 | 7.1 % | 2 | 7.1 % | 9 | 32.1 % | 8 | 28.5 % |
| >20 | 3 | 10.7 % | 2 | 7.1 % | 3 | 10.7 % | 3 | 10.7 % |
| (30-40) | 5 | 17.9 % | 11 | 39.3 % | 9 | 32.10% | 7 | 25.0 % |
| (60-90) | 2 | 7.10 % | 9 | 32.1 % | 10 | 35.7 % | 13 | 46.4 % |
| (100-120) | 0 | 0 | 0 | 0 | 4 | 14.3 % | 2 | 7.1 % |

**Table A3**: Generalized Estimating Equation Models Predicting Likelihood of Supplement Use and Exercise from Time

| **Variable** | **Predictor** | **Estimate** | **SE** | **Wald χ2** | **OR** | **95% CI** | **p-value** |
| --- | --- | --- | --- | --- | --- | --- | --- |
| Multivitamin | Intercept | -0.719 | 0.307 | 5.49 | 0.49 | 0.27 – 0.89 | 0.019* |
| Multivitamin | Time | 0.902 | 0.243 | 13.81 | 2.46 | 1.53 – 3.97 | <0.001* |
| Vitamin D | Intercept | -0.226 | 0.315 | 0.52 | 0.8 | 0.43 – 1.48 | 0.472 |
| Vitamin D | Time | 0.651 | 0.223 | 0.52 | 1.92 | 1.24 – 2.97 | 0.004 |
| Vitamin B12 | Intercept | -0.185 | 0.311 | 0.36 | 0.83 | 0.45 – 1.53 | 0.551 |
| Vitamin B12 | Time | 0.584 | 0.216 | 7.30 | 1.79 | 1.17 – 2.74 | 0.007 |
| Calcium | Intercept | -1.381 | 0.331 | 17.38 | 0.25 | 0.13 – 0.48 | <0.001* |
| Calcium | Time | 0.475 | 0.170 | 7.77 | 1.61 | 1.15 – 2.24 | 0.005* |
| Iron | Intercept | -0.935 | 0.315 | 8.79 | 0.39 | 0.21 – 0.73 | 0.003* |
| Iron | Time | 0.678 | 0.189 | 12.90 | 1.97 | 1.36 – 2.85 | <0.001* |
| Protein | Intercept | -1.023 | 0.3136 | 10.60 | 0.36 | 0.19 – 0.66 | 0.001 |
| Protein | Time | 0.070 | 0.1555 | 0.20 | 0.07 | 0.79 – 1.45 | 0.652 |
| Exercise | Intercept | -0.261 | 0.333 | 0.62 | 0.77 | 0.40 – 1.48 | 0.433 |
| Exercise | Time | 0.986 | 0.267 | 13.63 | 2.68 | 1.59 – 4.53 | <0.001 |

**Table A4.** Changes of vitamin D & B12, iron, & total protein concertation in blood over 12M follow-up

| **Clinical**  **data** | **Ref. Range** | **Baseline** | | **3 months** | | **6 months** | | **12 months** | |
| --- | --- | --- | --- | --- | --- | --- | --- | --- | --- |
|  |  | M | SD | M | SD | M | SD | M | SD |
| Total protein | 60-80 | 76.36 | 4.64 | 72.86 | 4.99 | 72.44 | 8.13 | 75.46 | 5.48 |
| Vitamin D | 50 – 175 | 33.94 | 16.04 | 76.34 | 37.37 | 80.20 | 44.35 | 84.04 | 41.98 |
| Vitamin B12 | 156 - 677 | 256.2 | 86.38 | 289.8 | 155.4 | 279.7 | 244.53 | 240.57 | 69.29 |
| Iron | 11 -31.3 | 12.20 | 3.82 | 13.82 | 4.30 | 14.76 | 4.55 | 13.66 | 4.61 |


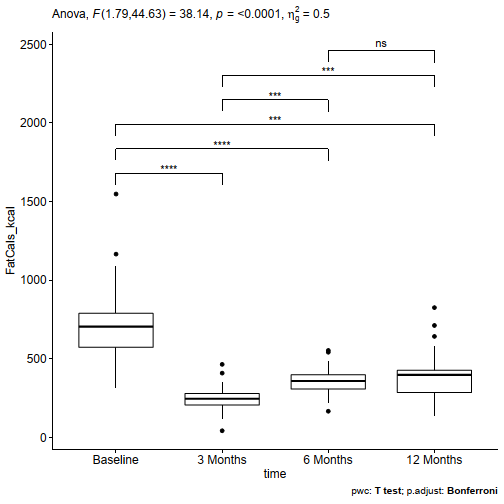

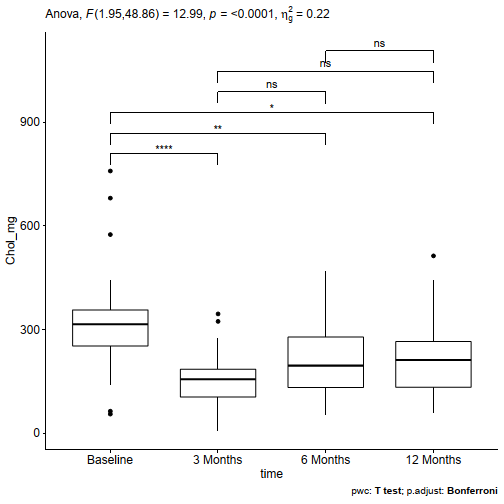


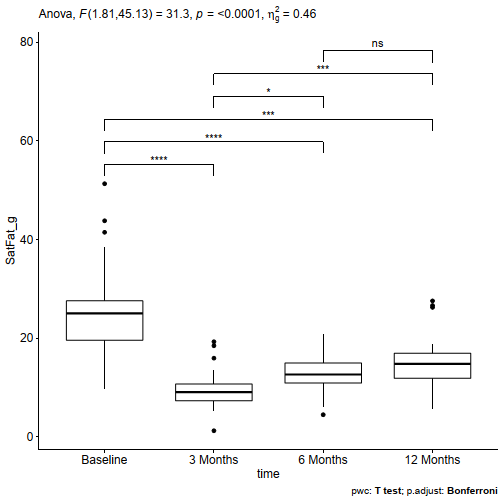

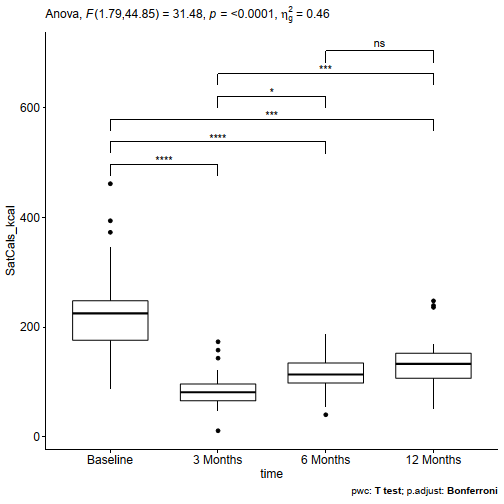


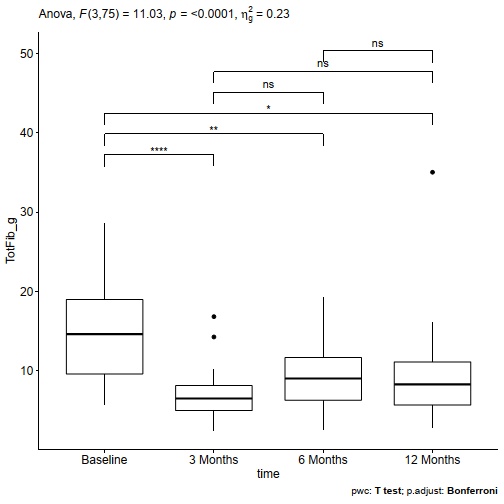

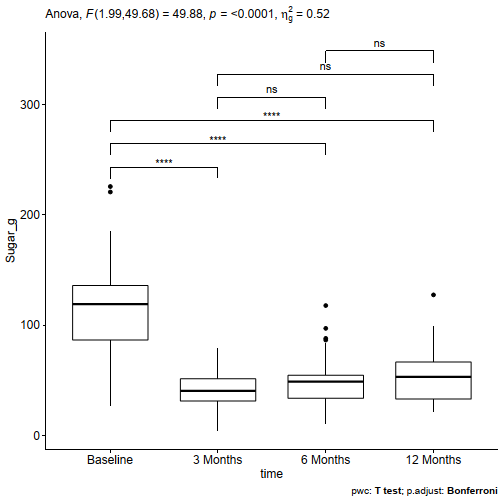


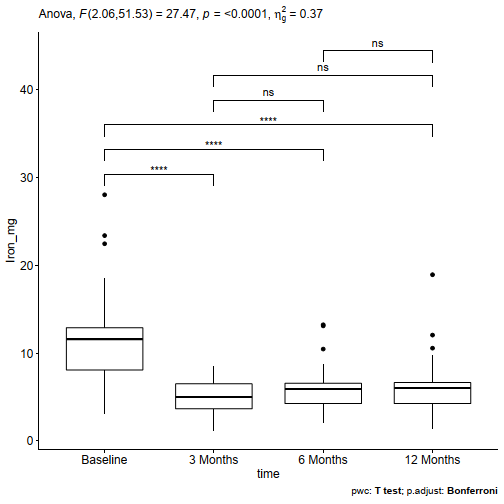

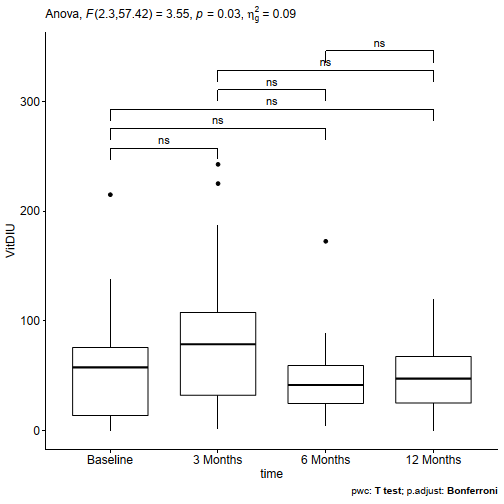


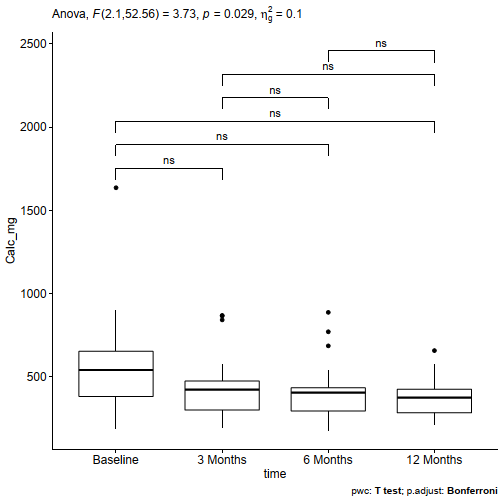

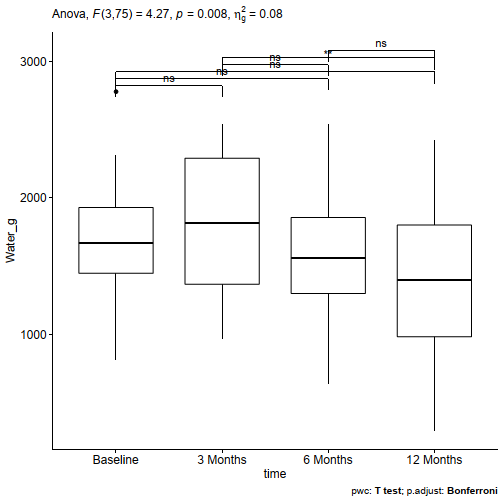


**Figure A1**: Macronutrients and micronutrients values in participants over one year follow-up from 24h dietary recall

| **Table A5:**  Food Frequency Questionnaire of participants over 12 M follow-up | | | | | | | | | | | | |  | |
| --- | --- | --- | --- | --- | --- | --- | --- | --- | --- | --- | --- | --- | --- | --- |
|  | **Baseline**  **(No) %** | | | **3 months**  **(No) %** | | | **6 months**  **(No) %** | | | **12 months**  **(No) %** | | | ***P* < 0.001**** | |
| **Nutrients** | **Sometime** | **Often** | **Always** | **sometime** | **Often** | **Always** | **Sometime** | **Often** | **Always** | **Sometime** | **Often** | **Always** |  |  |
| Fruit | (11) 39.3 | (7) 25.0 | (6) 21.4 | (11) 39.3 | (4) 14.3 | (9) 32.1 | (8) 28.6 | (11) 39.3 | (6) 21.4 | (7) 25.0 | (13) 48.4 | (8) 28.6 | 0.072 |  |
| vegetable | (8) 28.6 | (8) 28.6 | (7) 25.0 | (6) 21.4) | (9) 32.1 | (10) 36.7 | (10) 35.7 | (4) 14.3 | (12) 42.9 | (9) 32.1 | (6) 21.4 | (10) 35.7 | 0.365 |  |
| Dairy | (6) 21.4 | (8) 28.6 | (9) 32.1 | (6) 21.4 | (8) 28.6 | (10) 36.7 | (10) 35.7 | (6) 21.4 | (9) 32.1 | (7) 25.0 | (5) 17.9 | (12) 42.9 | 0.937 |  |
| Sweetened Drinks | (8) 28.6 | (6) 21.4 | (5) 17.9 | (1) 3.6 | 0 | 0 | 0 | (1) 3.6 | 0 | (1) 3.6 | (1) 3.6 | (1) 3.6 | <0.001** |  |
| Sweet Food | (10) 35.7 | (9) 32.1 | (4) 14.3 | (3) 10.7 | (1) 3.6 | 0 | (6) 21.4 | (2) 7.1 | (1) 3.6 | (9) 32.1 | (3) 10.7 | 0 | <0.001** |  |
| Snacks | (13) 46.4 | (3) 10.7 | (3) 10.7 | (4) 14.3 | (5) 17.9 | (2) 7.1 | (7) 25.0 | (5) 17.9 | (1) 3.6 | (12) 42.9 | (5) 17.9 | (1) 3.6 | 0.126 |  |
| Pastries | (11) 39.3 | (5) 17.9 | (3) 10.7 | (2) 7.1 | 0 | 0 | (6) 21.4 | (1) 3.6 | 0 | (12) 42.9 | (2) 7.1 | (1) 3.6 | <0.001** |  |
| Processed Meats | (5) 17.9 | (3) 10.7 | 0 | (2) 7.1 | 0 | 0 | (1) 3.6 | (2) 7.1 | 0 | (4) 14.3 | 0 | 0 | 0.010* |  |
| Whole wheat products | (4) 14.3 | (10) 35.7 | (8) 28.6 | (12) 42.9 | (6) 21.4 | (6) 21.4 | (8) 28.6 | (10) 36.7 | (5) 17.9 | (5) 17.9 | (10) 35.7 | (6) 21.4 | 0.007** |  |
| Fermented Yogurt | (5) 17.7 | (3) 10.7 | (4) 14.3 | (6) 21.4 | (3) 10.7 | (2) 7.1 | (6) 21.4 | (6) 21.4 | (3) 10.7 | (8) 28.6 | 0 | (3) 10.7 | 0.299 |  |
| Pickles | (5) 17.9 | (3) 10.7 | (1) 3.6 | 0 | (1) 3.6 | 0 | (7) 25.0 | (1) 3.6 | (1) 3.6 | (8) 28.6 | (2) 7.1 | 0 | 0.007** |  |
| Sea food | (11) 39.3 | (3) 10.7 | (2) 7.1 | (6) 21.4 | (3) 10.7 | (2) 7.1 | (9) 32.1 | (6) 21.4 | (3) 10.7 | (14) 50.0 | (4) 14.3 | (1) 3.6 | 0.156 |  |
| Chia / Flexed seeds | (3) 10.7 | (1) 3.6 | 0 | (2) 7.1 | 0 | 0 | (1) 3.6 | (1) 3.6 | 0 | (2) 7.1 | 0 | (1) 3.6 | 0.427 |  |
| Tea & Coffee | (2) 7.1 | (3) 10.7 | (20) 71.0 | (5) 17.9 | (2) 7.1 | (10) 35.7 | (5) 17.9 | (3) 10.7 | (14) 50.0 | (1) 3.6 | (9) 32.1 | (15) 53.6 | 0.006** |  |
| 4 cups of water everyday | (3) 10.7 | (2) 7.1 | (21) 75.0 | (2) 7.1 | (2) 7.1 | (23) 82.1 | (1) 3.6 | (7) 25.0 | (18) 64.3 | (1) 3.6 | (5) 17.9 | (20) 71.4 | 0.279 |  |
| fast food or restaurant | (12) 42.9 | (3) 10.7 | (7) 25.0 | (2) 7.1 | (1) 3.6 | (1) 3.6 | (8) 28.6 | (2) 7.1 | (1) 3.6 | (13) 48.4 | (1) 3.6 | (1) 3.6 | <0.001** |  |
| 3 main meals everyday | (6) 21.4 | (4) 14.3 | (4) 14.3 | (5) 17.9 | (5) 17.9 | (11) 39.3 | (6) 21.4 | (7) 25.0 | (10) 35.7 | (8) 28.6 | (7) 25.0 | (8) 28.6 | 0.013* |  |
| 3 snacks everyday | (7) 25.0 | (4) 14.3 | (4) 14.3 | (2) 7.1 | (4) 14.3 | (12) 42.9 | (11) 39.3 | (6) 21.4 | (7) 25.0 | (10) 35.7 | (4) 14.3 | (6) 21.4 | 0.024* |  |
